# Applicability of indocyanine green fluorescence tracing with subserosal dye injection in laparoscopic lymph node dissection for gastric cancer: a preliminary study

**DOI:** 10.20452/wiitm.2024.17898

**Published:** 2024-08-08

**Authors:** Zhangwei Yang, Pengwei Wang, Dandan Bao, Yi­‐Ren Hu, Senrui Chen, Yi Huang, Pingting Li

**Affiliations:** Department of General Surgery, Wenzhou People’s Hospital, Wenzhou, China; Wenzhou Medical University, Wenzhou, China

**Keywords:** fluorescence, indocyanine green, laparoscopic distal gastrectomy, lymph node dissection

## Abstract

**INTRODUCTION:**

Gastric cancer is a common malignancy of the digestive system, primarily metastasizing to the lymph nodes. Adequate lymph node dissection is crucial for accurate staging and improving patient prognosis. However, the complex distribution of lymph nodes around the stomach poses a significant challenge for thorough dissection.

**AIM:**

This study aimed to evaluate the efficacy and safety of laparoscopic radical gastrectomy guided by indocyanine green (ICG) fluorescence imaging with subserosal ICG injection in lymph node dissection for gastric cancer.

**MATERIALS AND METHODS:**

This retrospective study included 20 patients who underwent distal gastrectomy at the Wenzhou People’s Hospital between January 2020 and December 2022. Of them, 10 patients underwent conventional laparoscopic surgery, and the other 10 underwent laparoscopic surgery guided by ICG fluorescence imaging. The 2 groups were propensity-matched for sex, age, and preoperative cancer stage. General patient characteristics, lymph node dissection data, and perioperative outcomes, such as procedure time, blood loss, and postoperative complications, were compared between the groups.

**RESULTS:**

The total number of dissected lymph nodes was higher in the fluorescence-guided group than in the conventional surgery group, particularly in stations No. 6, 7, 8, 9, and 11p (all *P* <⁠0.001). There were no significant differences between the 2 groups in terms of procedure time, blood loss, and the incidence of postoperative complications.

**CONCLUSIONS:**

This preliminary study demonstrates that the use of ICG fluorescence imaging in minimally invasive radical gastrectomy can significantly increase the number of dissected lymph nodes without increasing the surgical risk.

## INTRODUCTION 

Gastric cancer is one of the most common malignant tumors of the digestive tract, ranking as the second leading cause of cancer-related death worldwide. In China, the incidence of gastric cancer remains high.^1^^; ^^2^^; ^^3^ Lymph node metastasis is a primary mode of gastric cancer spread, making thorough lymph node dissection critical for accurate staging and improving patient prognosis. Studies have shown that more extensive lymph node dissection not only aids in precise staging but also improves long-term outcomes of patients.^4^^; ^^5^^; ^^6^^; ^^7^^; ^^8^^; ^^9^^; ^^10^ International guidelines of the Union for International Cancer Control and the National Comprehensive Cancer Network recommend that at least 15 lymph nodes be dissected during radical gastrectomy for gastric cancer.^10^

However, the complex vascular and lymphatic networks around the stomach pose significant challenges for lymph node dissection during laparoscopic surgery, especially in patients with a higher body mass index. In cases of advanced gastric cancer, sufficient lymph node dissection using a minimally invasive approach is challenging. Achieving efficient and precise lymph node retrieval without increasing the risk for intraoperative complications is difficult, particularly for inexperienced surgeons.

Near-infrared fluorescence imaging with indocyanine green (ICG) has emerged as a novel surgical navigation technique. Recent studies have demonstrated that when ICG is locally injected around the primary gastric tumor, it is rapidly absorbed by the draining lymphatic system and remains there for a prolonged period.^11^^; ^^12^ Using near-infrared imaging systems, surgeons can clearly delineate the lymph node regions around the stomach in real-time, which allows them to distinguish lymph nodes from blood vessels, nerves, and fatty tissues. This significantly reduces the difficulty of lymph node dissection during laparoscopic gastric cancer surgery, improving both efficiency and accuracy.^13^^; ^^14^^; ^^15^^; ^^16^^; ^^17^

## AIM

This study aimed to compare the efficacy and safety of ICG fluorescence–guided laparoscopic distal gastrectomy with subserosal dye injection with conventional laparoscopic distal gastrectomy in lymph node dissection.

## MATERIALS AND METHODS 

### Patients 

This retrospective study included 20 patients, 10 of whom underwent indocyanine green (ICG) fluorescence–guided laparoscopic distal gastrectomy with subserosal dye injection, while the other 10 underwent conventional laparoscopic distal gastrectomy. All patients were treated at the Wenzhou People’s Hospital between January 2020 and December 2022. The groups were propensity-matched for age, sex, and preoperative cancer stage to ensure intergroup comparability. Initially, 36 patients who underwent distal gastrectomy for gastric cancer within the study period were considered. Propensity score matching was performed to select 10 patients for each group from among the 36 patients, aiming to achieve similar baseline characteristics. The collected data included basic patient characteristics (TABLE 1), lymph node dissection data, and perioperative outcomes (including procedure time, blood loss, postoperative complications, and postoperative staging) (TABLE 2).

**TABLE 1:** General patient characteristics

Variable		ICG fluorescence–guided LDG (n = 10)	Conventional LDG (n = 10)	*P *value
Age, y, mean (SD)		58 (11.5)	55 (11.4)	0.91
Men / women, n		4/6	4/6	1
Tumor site, n	Antrum	7	6	0.83
	Angle	3	4	0.75
Preoperative clinical stage (AJCC 8th ^32^ ), n	IA	2	1	0.59
	IB	3	4	0.75
	IIA	2	2	1
	IIB	2	3	0.69
Negative lymph nodes on preoperative imaging, n		5	4	0.78
Body mass index, kg/m^2^, mean (SD)		23.5 (1.8)	23.3 (2)	0.86
Tumor size, cm, mean (SD)		2.1 (0.8)	2.2 (0.9)	0.74
Neoadjuvant chemotherapy, n		2	2	1
Pathological type, n	Adenocarcinoma	7	8	0.61
	Signet ring cell carcinoma	2	1	0.31
	Mucinous adenocarcinoma	1	1	1

Abbreviations: AJCC, American Joint Committee on Cancer, ICG, indocyanine green; LDG, laparoscopic distal gastrectomy

**TABLE 2:** Perioperative outcomes and lymph node dissection data

Variable		ICG fluorescence–guided LDG (n = 10)	Conventional LDG (n = 10)	*P *value
Procedure time, min, mean (SD)		227 (26)	215 (14)	0.24
Intraoperative blood loss, ml, mean (SD)		75 (13)	79 (12)	0.5
Total lymph nodes dissected, n, mean (SD)		28.7 (5.4)	19.6 (4.7)	<0.001
Cases with <15 lymph nodes dissected		0	0	N/A
Station No. 1	Total dissected, n, mean (SD)	2.22 (0.67)	2.04 (0.83)	0.62
	Positive, n, mean (SD)	0	0	N/A
Station No. 3	Total dissected, n, mean (SD)	3.08 (1.49)	2.64 (0.76)	0.45
	Positive, n, mean (SD)	0	0	N/A
Station No. 4	Total dissected, n, mean (SD)	2.83 (0.96)	2.42 (0.99)	0.39
	Positive, n, mean (SD)	0	0	N/A
Station No. 5, 12a	Total dissected, n, mean (SD)	5.16 (0.6)	4.86 (0.95)	0.43
	Positive, n, mean (SD)	1.18 (0.64)	0.99 (0.42)	0.48
Station No. 6	Total dissected, n, mean (SD)	8.26 (1.13)	6 (0.84)	<0.001
	Positive, n, mean (SD)	1.96 (0.19)	1.53 (0.21)	<0.001
Station No. 7, 8, 9, and 11p^a^	Total dissected, n, mean (SD)	11.98 (2.55)	7.63 (2.39)	<0.001
	Positive, n, mean (SD)	4.07 (0.57)	2.98 (0.41)	<0.001
Complications according to the Clavien–Dindo classification,^33^ n				
Grade I		3	2	1
Grade II		3	1	0.55
Grade IIIa		0	0	N/A
Grade IIIb		0	0	N/A
Grade IV		0	0	N/A
Grade V		0	0	N/A
Postoperative staging (AJCC 8th^32^), n				
IA		2	1	0.59
IB		3	4	0.75
IIA		2	2	1
IIB		2	3	0.69
IIIA		1	0	0.29

a Due to the fact that lymph nodes in stations 7, 8, 9, and 11p are all located around the celiac trunk, they are collectively dissected as a single group during lymphadenectomy for gastric cancer. Therefore, these stations were combined into a single category for this study; Abbreviations: N/A, not applicable; others, see TABLE 1

### Surgical method

**FIGURE 1:**
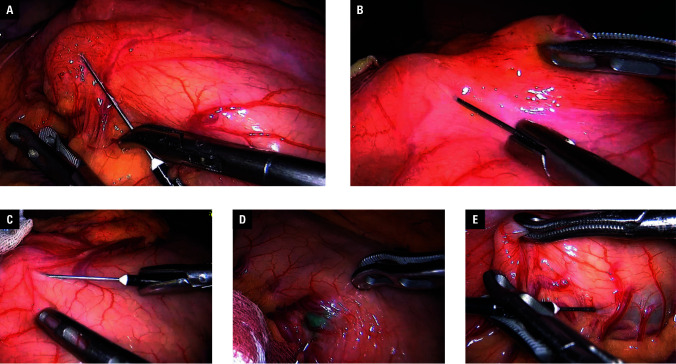
Injection sites of indocyanine green for fluorescence imaging

**FIGURE 2:**
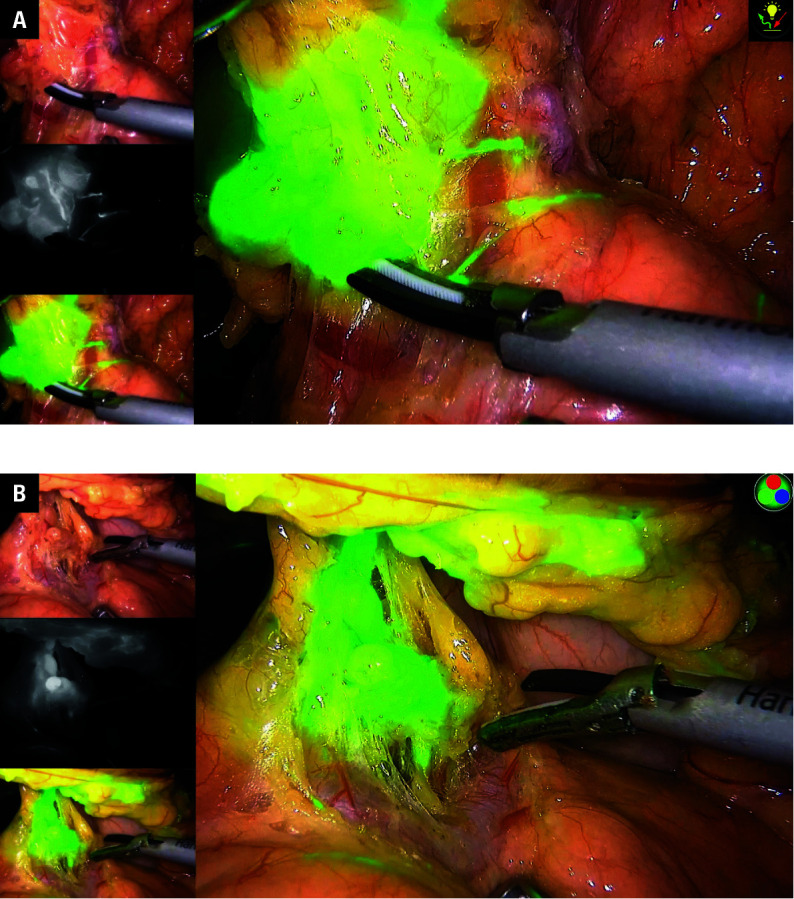
Visualization of lymphatic structures after indocyanine green injection, showing enhancement and delineation achieved through fluorescence

All patients were placed in a supine position, with legs apart. A viewing port was established below the umbilicus, and operational ports were set up at 2 cm below the costal margin, on the left and right anterior axillary lines, and on the upper abdominal wall, following the conventional 5-port method. After confirming a lack of significant metastases in the abdominal cavity, ICG was injected subserosally in the ICG group at specific sites (the first branch of the left gastroepiploic artery, the first branch of the right gastroepiploic artery near the midpoint of the greater curvature, the gastric angle, the first and second branches of the left gastric artery, and the first branch of the right gastric artery). The injection concentration was 0.2–0.5 mg/ml, with 1.5 ml per site.^18^ The surgical procedure started immediately after the injection to ensure that by the time the dissection phase began, the dye had been adequately absorbed and provided optimal fluorescence imaging. Guided by ICG fluorescence, distal gastrectomy and D2 lymph node dissection were performed following the Japanese Gastric Cancer Treatment Guidelines.^19^ Lymph node dissection was performed in the following order: station No. 4, No. 6, No. 7, 8, 9, and 11p, No. 5, No. 12a, No. 3, and No. 1. In the control group, the procedure followed the same protocol, without ICG injection. In both groups, surgeries were performed by the same experienced surgical team, adhering to strict quality control measures. Periprocedural images are shown in FIGURE 1 and FIGURE 2.

**A** – the first right epiploic artery; **B** – the first right gastric artery; **C** – the gastric angle; **D** – the first and second branches of the left gastric artery; **E** – the first left epiploic artery (near the midpoint of the greater gastric curvature)

**A** – station No. 6 lymph nodes; **B** – stations No. 7, 8, 9, and 11p lymph nodes

### Ethics statement

This study protocol was reviewed and approved by the Ethics Committee of Wenzhou People’s Hospital (ky-2023-244). All patients signed an informed consent form. The images presented in the article were approved for publication by both the patients and the Ethics Committee.

### Statistical analysis

Data analysis was conducted using the R software (The R Foundation for Statistical Computing, Vienna, Austria). Quantitative data are expressed as mean (SD) and were compared using the analysis of variance. Qualitative data are presented as numbers and were compared using the χ^2^ test. A *P* value below 0.05 was considered significant. Propensity matching was performed using the R software “Matching” package.

## RESULTS

The total number of dissected lymph nodes in the fluorescence-guided group was higher than in the control group (mean [SD], 28.7 [5.4] vs 19.6 [4.7]; *P* <⁠0.001). In particular, the number of dissected lymph nodes was significantly higher in stations No. 6 (mean [SD], 8.26 [1.13] vs 6 [0.84] in the fluorescence-guided vs conventional surgery group, respectively) and No. 7, 8, 9, and 11p (mean [SD] 11.98 [2.55] vs 7.63 [2.39] in the fluorescence-guided vs conventional surgery group, respectively). The overall number of positive nodes was also higher in the fluorescence-guided group as compared with the control group (mean [SD], 4.07 [0.57] vs 2.98 [0.41]; *P* <⁠0.001). There was no significant difference in the number of dissected lymph nodes in stations No. 1, 3, 4, 5, and 12a. Also, no significant differences were found between the 2 groups in terms of procedure time, blood loss, and the incidence of postoperative complications (TABLE 2).

## DISCUSSION

The application of ICG fluorescence imaging in medical research dates back to the 1960s. It was initially used for evaluating cardiac and liver function. In recent years, ICG has been increasingly used for sentinel lymph node mapping and tissue perfusion assessment, yielding favorable clinical outcomes.^11^^; ^^12^^; ^^17^ With the introduction of ICG-labeled near-infrared laparoscopic systems, the use of ICG in laparoscopic surgeries has expanded significantly. The laparoscopic application of ICG includes intravenous injection for tissue perfusion evaluation as well as local injection around tumors for tumor localization and lymph node navigation. Given the rich vascularization and complex lymphatic drainage of the stomach, lymph node dissection in gastric cancer surgery has always been challenging, impacting patient prognosis. Fluorescence-guided lymph node dissection has thus become a focus of research,^20^^; ^^21^^; ^^22^ offering new solutions for improving the accuracy of lymph node dissection and patient outcomes.

Currently, the effectiveness of ICG fluorescence imaging in enhancing lymph node retrieval in minimally invasive gastric cancer surgery is still debated. Some studies, such as the one by Lan et al,^23^ reported no significant increase in total lymph node yield in the ICG group as compared with the non-ICG group. However, other works, including those by Kwon et al^24^ and Cianchi et al,^25^ found that ICG fluorescence–guided minimally invasive gastrectomy significantly improved lymph node retrieval without compromising short-term outcomes. Differences in these findings may be attributed to variations in ICG injection methods, timing, and concentrations. Huang et al^18^ showed that submucosal ICG injection around the tumor at 3, 6, 9, and 12 o’clock positions 24 hours before surgery resulted in achieving good intraoperative imaging results. Based on these findings, Chen et al^26^ conducted the first prospective randomized controlled trial to evaluate the clinical efficacy of ICG fluorescence–guided lymph node dissection in laparoscopic gastric cancer surgery. They demonstrated that ICG guidance allows for more effective lymph node retrieval without increasing the risk of postoperative complications, providing valuable evidence for the use of ICG in gastric cancer surgery. Patti et al^27^ recommended routine use of ICG tracing technology in gastric cancer surgery. These studies offer important guidance and references for further promotion and optimization of ICG fluorescence imaging application in minimally invasive gastric cancer surgery.

ICG can be administered in 2 ways to guide lymph node dissection for gastric cancer: preoperative endoscopic submucosal injection or intraoperative laparoscopic subserosal injection. Previous retrospective studies and a randomized controlled trial indicated that preoperative submucosal ICG injection effectively traced perigastric lymph nodes, significantly increasing lymph node yield without increasing the risk of surgical complications.^28^^; ^^29^^; ^^30^ Traditional approaches prefer preoperative submucosal ICG injection; however, recent research confirmed that intraoperative subserosal ICG injection yields similar lymph node retrieval results and is associated with a comparable surgical burden. Moreover, subserosal injection is more convenient and associated with lower economic and psychological burdens, which makes it the recommended method.^31^

This study shows that subserosal ICG injection significantly enhances the safety and efficacy of lymph node dissection. A significantly higher total number of dissected lymph nodes and positive nodes was observed in the fluorescence-guided group, as compared with the conventional surgery group. Particularly in stations No. 7, 8, 9, and 10, fluorescence imaging provided clearer visibility, facilitating more thorough dissection. These lymph node groups are critical for D2 dissection, and thorough dissection is crucial for improving patient prognosis. Specifically, dissection of station No. 7, 8, and 9 lymph nodes is challenging in conventional surgery, but is improved in fluorescence-guided conditions, increasing thoroughness. The study also showed significant improvements in station No. 6 lymph node dissection in the fluorescence-guided group. In patients with distal gastric cancer, station No. 6 lymph node dissection in conventional surgery often results in injuries to the transverse mesocolon or middle colic artery, leading to colonic ischemia requiring resection. Under fluorescence imaging, drainage areas of the station No. 6 lymph nodes and the transverse colon can be clearly distinguished, which aids in accurate dissection and significantly improves safety and efficacy of the procedure. Additionally, no significant differences in perioperative complications were observed between the groups, indicating that ICG fluorescence–guided lymph node dissection does not increase surgical risk while enhancing lymph node retrieval. This has positive implications for perioperative recovery and long-term outcomes of patients.

This study has several limitations. First, it is retrospective, nonrandomized, and was performed in a single center, which limits generalizability of the results. Second, long-term survival analysis of the patients was not performed; hence, further observation is needed to determine whether the use of ICG technology can improve long-term survival rates. Additionally, during the subserosal injection of ICG, there were occasional cases of leakage, which could compromise the quality of lymph node imaging. The operability of this technique needs further improvement.

## CONCLUSIONS

Overall, ICG fluorescence imaging technology with subserosal dye injection in minimally invasive radical gastrectomy shows broad application prospects, significantly increasing lymph node retrieval and ensuring surgical safety. This approach is expected to improve surgical precision and patient outcomes. Future research and experiences will help further refine this technology and provide more reliable treatment options for minimally invasive gastric cancer surgery.
